# The evolution of personalized stroke thrombectomy

**DOI:** 10.3389/fsurg.2025.1590146

**Published:** 2025-07-29

**Authors:** Emmanuel O. Mensah, Yew-Weng Fong, Sandeep Muram, Christopher S. Ogilvy, Philipp Taussky

**Affiliations:** ^1^ Neurosurgical Service, Beth Israel Deaconess Medical Center and Harvard Medical School, Boston, MA, United States; ^2^ Department of Neurosurgery, Cathay General Hospital, Taipei, Taiwan

**Keywords:** stroke thrombectomy, personalized medicine, large vessel occlusion, endovascular treatment, medium vessel occlusion

## Abstract

Stroke is a leading cause of disability and death worldwide, with acute ischemic stroke accounting for most cases. Mechanical thrombectomy is a widely accepted treatment modality in appropriately selected patients, demonstrating improved functional outcomes through safe and effective recanalization. However, traditional trials have focused on a narrow subset of patients, limiting its applicability to diverse populations who would otherwise benefit from thrombectomy. Advances in neurovascular imaging, device innovation, and procedural techniques are driving a paradigm shift toward personalized stroke thrombectomy. This review explores personalization strategies across various domains, including lesion-specific considerations such as medium vessel occlusions (MeVOs), basilar artery occlusions (BAOs), and tandem lesions, as well as patient-specific factors like infarct size, low NIHSS scores, advanced age, and unique biomarkers. Additionally, we discuss procedural innovations, such as tailored device use and alternative access strategies to address anatomical and clinical complexities. While substantial progress has been made, challenges remain in refining patient selection criteria, mitigating procedural risks, and ensuring equitable access to thrombectomy. Future directions include taking full advantage of advanced imaging modalities, incorporating biomarkers for personalized care, and optimizing thrombectomy devices to support the use of thrombectomy in underrepresented populations. Precision thrombectomy has the potential to be adapted to a broader spectrum of patients, improving outcomes and ultimately reducing the global burden of stroke.

## 1 Introduction

Stroke is a leading cause of permanent disability and death worldwide, representing a global health concern (1). Accounting for approximately 87% of all strokes (2), acute ischemic stroke (AIS) occurs secondarily to thrombosis, embolism, or systemic hypoperfusion, of which approximately 40% are large vessel occlusions (LVOs) (3). Mechanical thrombectomy has emerged as the gold standard for AIS treatment, restoring cerebral perfusion and improving functional outcomes through complete recanalization of occluded intracranial arteries. While landmark studies validated the safety and efficacy of thrombectomy in acute ischemic stroke, these studies selected patients according to stringent inclusion criteria, such as early presentation and small core infarcts (4–8). However, many patients affected by stroke deviate from this phenotype, presenting new challenges and opportunities for personalized approaches.

Advances in neurovascular imaging and iterative thrombectomy device development have improved patient selection and procedural safety. The evolution of stroke thrombectomy is shifting towards precision medicine, optimizing treatment strategies to patient- and lesion-specific factors, including vessel occlusion type, infarct size, collateralization status, patient age, comorbidities, and individual biomarkers. This review explores this paradigm with the aim to guide clinicians and researchers in advancing personalized approaches to thrombectomy to expand its eligibility to traditionally excluded patient groups.

## 2 Personalization by occluded vessel type

### 2.1 Large vessel occlusion

Personalized stroke thrombectomy in large vessel occlusion (LVO) focuses on tailoring treatment strategies based on patient-specific and stroke-specific factors to maximize outcomes, with initial trials focusing on patients in early time windows (≤6–≤12 h from last known well, LKW) (Table 1). The MR CLEAN trial first demonstrated the superiority of thrombectomy using second-generation stent retrievers, such as Solitaire® and Trevo®, over standard medical management, including intravenous thrombolysis (IVT) (4). Patients treated with thrombectomy showed significantly higher rates of functional independence (mRS 0–2 at 90 days: 32.6% vs. 19.1%) and smaller infarct volumes. These findings were corroborated by subsequent trials, which included LKW times of ≤6 h (SWIFT PRIME and EXTEND-IA) (5, 6), extending to ≤8 h (REVASCAT) (7), and finally ≤12 h (ESCAPE) (8), collectively leading to the HERMES meta-analysis (9). Time from LKW status was 6 h in SWIFT This analysis confirmed that thrombectomy nearly doubled the odds of achieving good functional outcomes (adjusted OR 2.49), emphasizing the importance of rapid intervention in a specific subgroup of patients. Following these results, thrombectomy was recommended as the preferred treatment for LVOs in the anterior circulation. The American Heart Association/American Stroke Association (AHA/ASA) provided a Class IA recommendation for patient inclusion criteria for thrombectomy, including baseline functional status (mRS 0–1), high ASPECTS (≥6), and NIHSS ≥6 for strokes in the anterior circulation (internal carotid artery and M1 segment of the middle cerebral artery) who present within 6 h of onset (10).

**Table 1:** Characteristics of RCTs investigating mechanical thrombectomy in large vessel occlusion in the early window.

Trial	MR CLEAN (4)	SWIFT PRIME (5)	EXTEND-IA (6)	REVASCAT (7)	ESCAPE (8)
Study characteristics					
Publication year	2015	2015	2015	2015	2015
Enrolment years	2010–2014	2012–2014	2012–2014	2012–2014	2013–2014
Country	Netherlands	Multi-national	Australia, New Zealand	Spain	Multi-national
Sample	500	196	70	206	315
Inclusion criteria					
Age	≥18	18–85, later changed to 18–80	≥18	18–80, later changed to 81–85 if ASPECTS >8	≥18
Time	0–6 h	0–6	0–6	0–8	0–12
Vessels	ICA, M1, M2, A1, A2	ICA, M1	ICA, M1, M2	ICA, M1	ICA, M1, M2
ASPECTS/Lesion volume	0–10	ASPECTS ≥6; volume <50 ml with mismatch	Volume <70 ml with mismatch	ASPECTS ≥7 (≥8 if age 81–85)	ASPECTS ≥6
NIHSS	≥2	8–29	–	≥6	≥5
Baseline mRS	–	≤1	≤1	≤1	–
TPA eligibility	Not required	Required	Required	Not required	Not required
Outcomes					
mTICI 2b, 3	75.4%	88.0%	86.2%	66.0%	72.4%
mRS 0–2	32.6% vs. 19.1%	60.2% vs. 35.5%	71% vs. 40%	43.7% vs. 28.2%	53% vs. 29.3%
Mortality	21% vs. 22%	12% vs. 9%	9% vs. 20%	18% vs. 15%	10% vs. 19%
sICH	7.7% vs. 6.4%	0% vs. 3%	0% vs. 6%	1.9% vs. 1.9%	3.6% vs. 2.7%
NNT	7.1	4	3.2	6.5	4.2

A1, A1 segment of the anterior cerebral artery (ACA); A2, A2 segment of the ACA; ASPECTS, Alberta Stroke Program Early CT Score; ICA, internal carotid artery; M1, M1 segment of the middle cerebral artery (MCA); M2, M2 segment of the MCA; mRS, modified Rankin score; mTICI, modified thrombolysis in cerebral infarction score; NIHSS, National Institutes of Stroke Scale; NNT, number needed to treat; sICH, symptomatic intracranial bleeding; TPA, tissue plasminogen activator.

**Figure 1:**
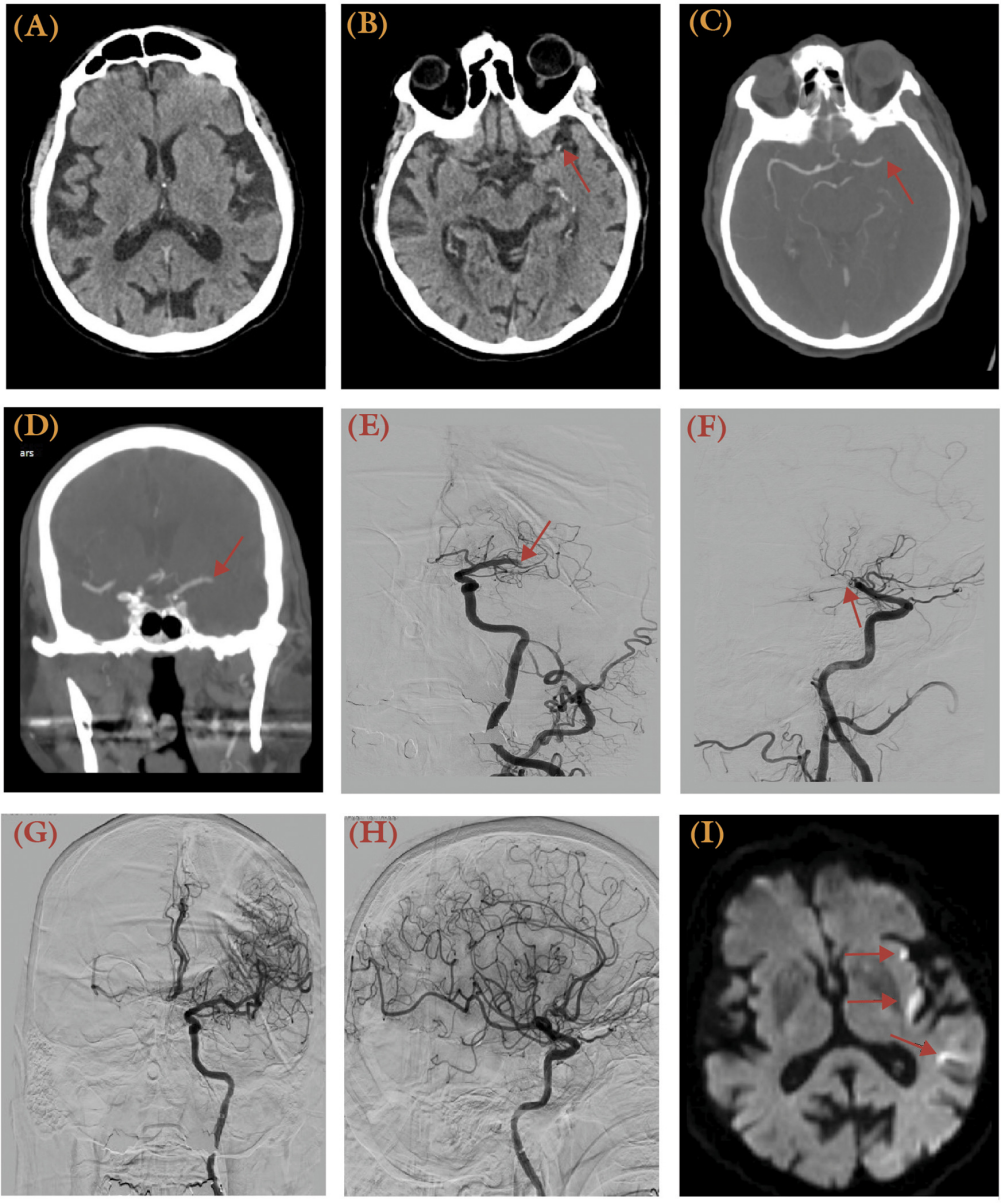
An 89-year-old male with a history of paroxysmal atrial fibrillation treated with 15 mg daily rivaroxaban, hypertension, and hyperlipidemia presented with right-sided weakness and aphasia, with the last known well 12 h prior. He had an NIH Stroke Scale (NIHSS) score of 16 and an ASPECTS score of 10. **(A)** No hypodense lesion was apparent from initial CT at presentation. **(B)** MCA dot sign was visible (arrow). **(C,D)** A computed tomography angiography (CTA) revealed a left distal M1 occlusion (arrow) with no distal perfusion. **(E,F)** Anteroposterior **(E)** and lateral **(F)** views of the digital subtraction angiography (DSA) illustrates the occlusion site at the left distal M1 (Arrow). **(G,H)** Post-thrombectomy, a TICI 3 recanalization was achieved using the ADAPT technique in anteroposterior **(G)** and lateral **(H)** views of the angiogram. **(I)** A post-thrombectomy MRI diffusion-weighted imaging (DWI) series revealed multiple punctate areas of restricted diffusion in the left insular and temporal regions, representing acute infarcts.

Personalized thrombectomy in the late time windows (6–24 h from LKW) represents a paradigm shift from time-based to tissue-based patient selection, driven by advanced imaging techniques (Table 2). Landmark trials such as DAWN and DEFUSE−3 demonstrated the efficacy of thrombectomy in patients with anterior circulation LVOs who had a small ischemic core but significant salvageable penumbra. The DAWN trial, which included patients up to 24 h post-stroke onset, showed a nearly fourfold increase in functional independence at 90 days (mRS 0–2 in 49% vs. 13% of the control group) (11), while DEFUSE-3 reported similar benefits for patients treated within 6–16 h (12). The results were transformative, with a number needed to treat (NNT) of just 3 to achieve improved functional outcomes. These findings led to revised guidelines supporting thrombectomy for selected late-window patients, emphasizing the importance of advanced imaging, such as CT perfusion or MRI, to identify ischemic core-penumbra mismatch. Recent meta-analyses, including the Analysis of Pooled Data from Randomized Studies of Thrombectomy More Than 6 Hours After Last Known Well (AURORA) collaboration, further solidified the benefit of thrombectomy across diverse subgroups, including older patients (age ≥80 years) and those with wake-up strokes (13). An illustrative case of an 89-year-old male presenting with National Institutes of Stroke Scale (NIHSS) score of 16 due to a left distal M1 occlusion, successfully recanalized using aspiration thrombectomy, is shown in Figure 1.

**Table 2:** Characteristics of RCTs investigating mechanical thrombectomy in large vessel occlusion in the late window.

Trial	DAWN (11)	DEFUSE-3 (12)	MR CLEAN-LATE (17)
Study characteristics			
Publication year	2017	2018	2023
Enrolment years	2014–2017	2016–2017	2018–2022
Sample	206	182	502
Inclusion criteria			
Age	≥18	18–90	≥18
Time	6–24 h	6–16 h	6–24 h
Vessels	ICA, M1	ICA, M1	ICA, M1, proximal M2
ASPECTS	≥10	≥6	
Lesion/Infarct volume	<21 ml for age ≥80 and NIHSS ≥10 <31 ml for age <80 and NIHSS ≥10 31–51 ml for age (<80 and NIHSS ≥20	<70 ml, mismatch penumbra/core ratio ≥1.8 and absolute penumbra vol ≥15 ml	–
NIHSS	≥10 and ≥20	≥6	≥2
Baseline mRS	≤1	≤2	≤2
TPA eligibility	Ineligible for, or failed, IV tPA	Not required	Not required
Outcomes			
mTICI 2b or 3	72% (90%)	76%	73%
mRS 0–2	49% vs. 13%	45% vs. 17%	39% vs. 34%
Mortality	19% vs. 18%	14% vs. 26%	24% vs. 30%
sICH	6% vs. 3%	7% vs. 4%	7% vs 2%
NNT	2.8	3.6	–

ASPECTS, Alberta Stroke Program Early CT Score; ICA, internal carotid artery; M1, M1 segment of the middle cerebral artery (MCA); M2, M2 segment of the MCA; mRS, modified Rankin score; mTICI, modified thrombolysis in cerebral infarction score; NIHSS, National Institutes of Stroke Scale; NNT, number needed to treat; sICH, symptomatic intracranial bleeding; TPA, tissue plasminogen activator.

Collateralization status is the underlying phenomenon for this tissue-based approach, as it influences infarct progression and treatment outcomes. Collateral vessels provide alternative pathways for blood flow, sustaining ischemic penumbra and delaying core infarct expansion (14). Studies have shown that patients with robust collaterals, termed “slow progressors,” lose neurons at significantly lower rates compared to those with poor collaterals, or “fast progressors,” who experience rapid infarct growth (15, 16). The MR CLEAN-LATE trial demonstrated that thrombectomy for anterior circulation ischemic stroke is effective in the late window (6–24 h from LKW) when patients are selected based on collateral flow assessment via CTA. At 90 days, thrombectomy was associated with significantly better functional outcomes compared to best medical management alone, with a shift towards lower modified Rankin Scale (mRS) scores [adjusted common odds ratio (OR) 1.67; 95% CI 1.20–2.32] (17). These benefits persisted at 2 years, with the thrombectomy group maintaining improved functional outcomes (adjusted OR 1.41; 95% CI 1.00–1.99) (18). Importantly, all patients included had confirmed collateral flow, emphasizing its pivotal role in identifying viable ischemic penumbra and guiding late-window thrombectomy. While symptomatic intracranial hemorrhage (sICH) was more frequent in the thrombectomy group, all-cause mortality was not significantly different, and thrombectomy proved safe and effective for improving long-term outcomes.

### 2.2 Medium vessel occlusions

Medium vessel occlusion refers to distal intracranial arterial occlusions, typically 0.75–2 mm in diameter (19). These occlusions occur in M2, M3, or M4 branches of the MCA, A2 or A3 branches of the ACA, and P2 or P3 branches of the PCA (20). Additionally, some authors consider occlusions of the A1 and P1 segments as MeVOs (21). Occlusions of the M2 segment of the MCA may also be considered an LVO or MeVO depending on the dominance of the M2 branch, with dominant M2 branch occlusions categorized as LVOs and co-dominant or non-dominant M2 branch occlusions categorized as MeVOs (22). MeVOs, arising as either primary occlusions or secondarily to previous angiographic or thrombectomy procedures, are responsible for 25%–40% of all acute ischemic strokes (19, 23). Given the high risk of long-term disability associated with MeVOs in up to 50% of patients (24), mechanical thrombectomy should be offered to appropriately selected patients. However, several challenges to the use of mechanical thrombectomy for these lesions exist. By categorization, MeVOs represent a heterogenous group of conditions as characterized by the variability in occlusion sites and neurological deficits. These occluded vessels are also smaller, with thinner and more fragile walls, and are more prone to dissection, rupture, and vasospasm. The tortuosity of these distal vessels also impede navigation during mechanical thrombectomy (25–27). Despite the challenges, development of smaller mechanical thrombectomy devices, along with the effectiveness of intra-arterial fibrinolysis in just a third of these cases (19, 28), and reported efficacy of thrombectomy for MeVOs in retrospective studies (29), thrombectomy represents a valuable treatment modality for patients with MeVOs. As such, the 2019 AHA/ASA and Society for Neuro-interventional Surgery guidelines recommend thrombectomy for patients with M2 or M3 occlusions (class IIB) and M2 occlusions, respectively (10, 30).

**Figure 2:**
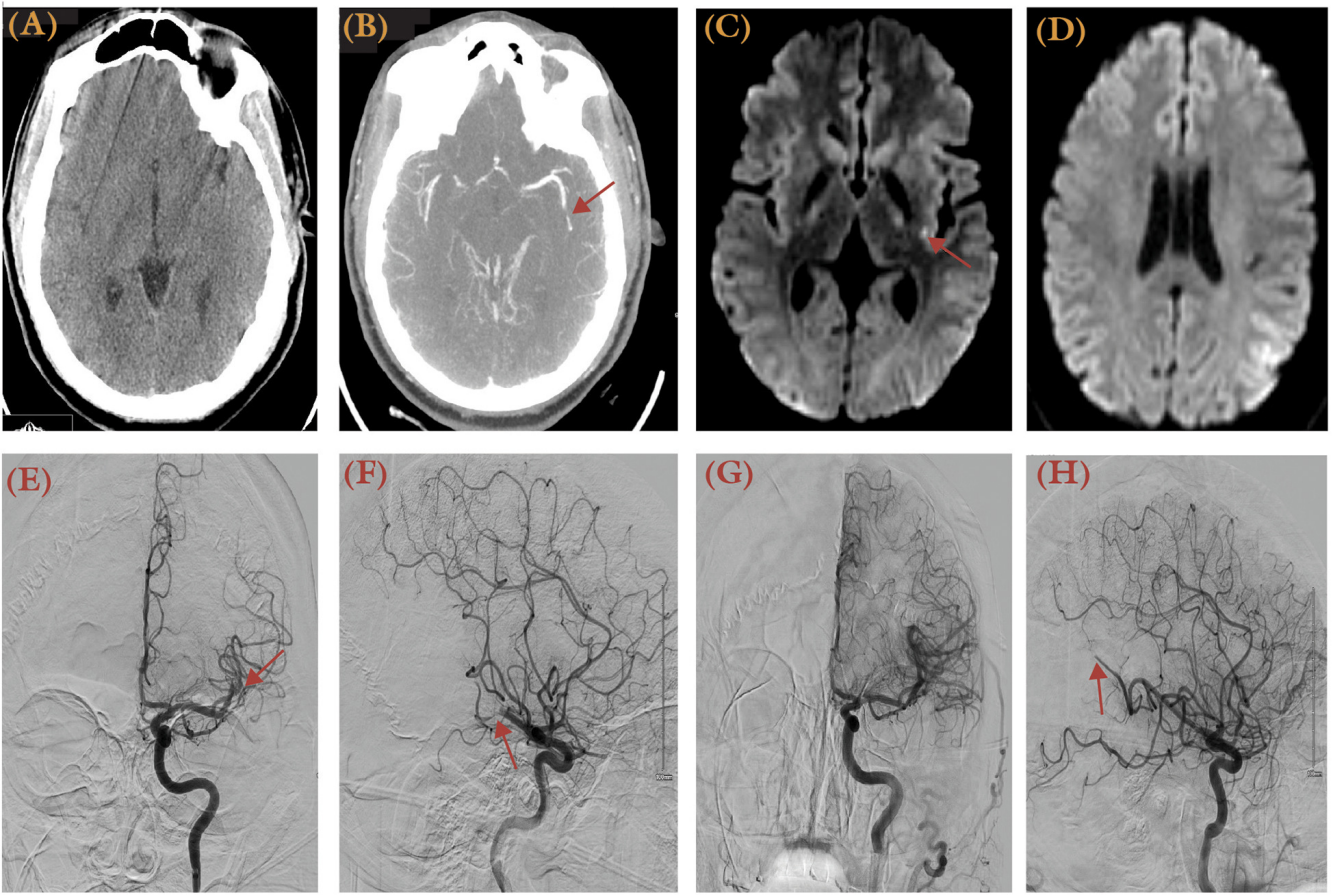
A 32-year-old obese male with a history of obesity and heart failure presented with aphasia, dysarthria, and right arm numbness, having been last known normal 1 h prior to presentation. His NIHSS score was 6. **(A)** Non-contrast brain CT showed an ASPECTS score of 10. **(B)** Brain CTA revealed an occluded left M2 vessel (arrow). **(C,D)** MRI DWI series revealed focal restrictions at left insular and parietal regions. Anterior-posterior **(E)** and lateral views **(F)** of DSA (left ICA injection) illustrated the site of occlusion as the M2 segment (arrow). **(G,H)** DSA after thrombectomy using the ADAPT technique demonstrated a TICI 2C recanalization. Distal occlusion at M3 segment was not pursued further.

The safety and efficacy of EVT for MeVOs in the MCA circulation has been documented. A single center study of 62% primary and 33% secondary heterogenous MeVO lesions reported significant reperfusion (mTICI 2b-3 in 83%) with observed 30% 90–day functional independence and 20% mortality rates (31). A patient level meta-analysis of M2 MCA occlusions from data in the HERMES Collaboration demonstrated superior outcomes in thrombectomy-treated patients (mRS 0–2 in 58.2%) compared to controls (39.7%), with maximal benefit in proximal and dominant M2 occlusions (32). A case of a 32-year-old female with NIHSS 6 due to a left M2 occlusion, treated successfully with the ADAPT technique achieving TICI 2C recanalization, is shown in Figure 2. For non-M2 MCA MeVOs, a recent meta-analysis showed limited benefit for thrombectomy over control in A2, M3, P2, P3, and P4 lesions, with good outcomes in 54.7% vs. 54.5% respectively, although not statistically significant (33).

In the posterior circulation, the TOPMOST trial demonstrated efficacy and safety of thrombectomy in P2 and P3 occlusions among IVT-ineligible patients with admission NIHSS ≥10 (34). Similarly, thrombectomy was shown to be feasible for A2 to A4 occlusions, primarily due to large artery atherosclerosis, with a low sICH rate of 2% (35). The PLATO study, which focused on posterior circulation MeVOs, observed greater NIHSS improvement and earlier clinical benefit in the thrombectomy group, despite no significant shift in 90-day mRS. However, this group also experienced higher rates of sICH and mortality, emphasizing the need to balance functional benefit with procedural risk. Stratified analysis suggested thrombectomy benefit among patients with baseline NIHSS >6 and no increase in adverse events. The limited value of mRS and NIHSS scores in posterior circulation strokes has also been reported, necessitating holistic assessments in functional status following treatment of these MeVOs (36).

Notably, two recent RCTs provide further clarity. The ESCAPE-MeVO trial, enrolling 530 patients with acute MeVOs (predominantly MCA branch occlusions), found no significant difference in 90-day functional independence (mRS 0–1 in 41.6% EVT vs. 43.1% control, *p* = 0.61) and reported higher mortality and sICH rates in the EVT group (37). Likewise, the DISTAL trial, evaluating EVT in distal ACA, MCA, and PCA occlusions, showed no significant improvement in mRS at 90 days with EVT over best medical therapy (common OR 0.9, *p* = 0.50), with comparable mortality and sICH rates between groups (38). As future trials continue to refine treatment indications, especially for patients with mild symptoms or distal occlusions, personalization will remain central to expanding the therapeutic frontier for MeVOs.

### 2.3 Basilar artery occlusions

Basilar artery occlusions are associated with high mortality rates despite treatment (39). Patients with these strokes have a varied presentation, with clinical symptoms including cranial nerve defects, dysarthria, dysphagia, gait ataxia, and hemiplegia that progresses to locked-in syndrome (39). These signs and symptoms are typically not accurately captured with the NIHSS score, requiring more appropriate endpoints for assessing patient functional status. To mitigate this, scoring systems like the POST-NIHSS score have been proposed. Imaging-based markers, like the pons-midbrain index, have also been explored, though they are not validated clinical severity scores (40, 41). Factors associated with favorable outcomes post- thrombectomy for BAOs include a low pretreatment NIHSS score, duration of symptom (<6 h), good collateralization status, the absence of an early pontine infarct, and thrombectomy technique. Proper identification of appropriate patients with BAO for thrombectomy is strongly supported by current guidelines (36, 42).

**Figure 3:**
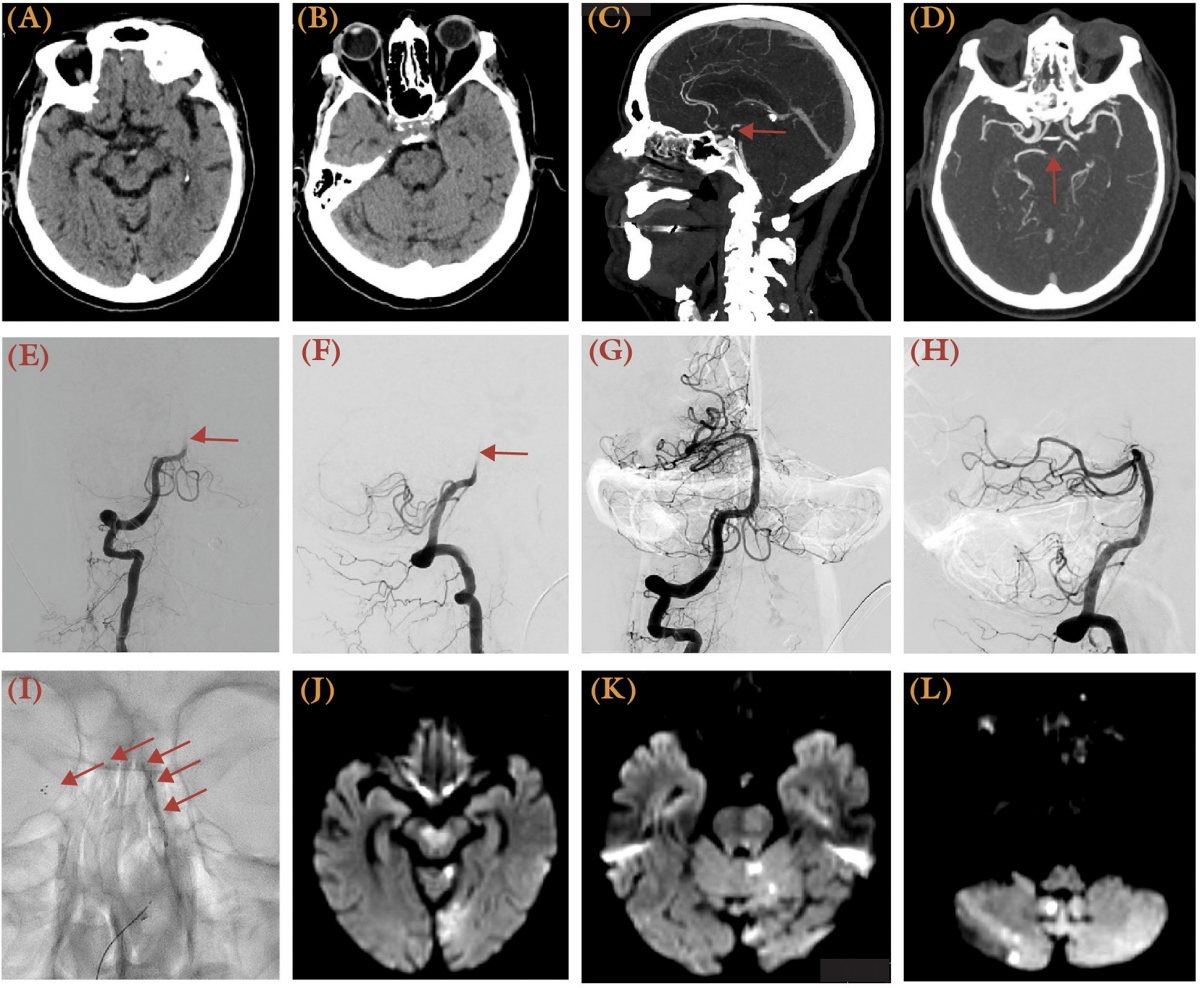
This 78-year-old female with a history of coronary heart disease, atrial fibrillation, hypertension, and hyperlipidemia experienced acute dizziness, gaze palsy, aphasia, and right-sided weakness, presenting with an NIHSS score of 28 and last known well 3.5 h prior to presentation. **(A,B)** Brain CT demonstrated possible hypodense over the bilateral midbrain and pons, with <50% involvement and the left cerebellum. Sagittal **(C)** and axial **(D)** views of brain CTA MIP images demonstrated an upper basilar occlusion (arrow). **(E,F)** Right vertebral artery injection DSA showed restriction of contrast flow at the vertebrobasilar junction (arrow) in both anterior-posterior **(E)** and lateral views **(F)** before mechanical thrombectomy. **(G,H)** A TICI 3 recanalization was achieved after two passes using the SOLUMBRA technique. **(I)** Unsubtracted x-ray illustrates the stent retriever, as delineated by arrows. **(J–L)** A follow-up MRI showed areas of restricted diffusion over the cerebellum, left occipital lobe, and brainstem.

Data from the BASILAR registry, including 1,254 patients, was the first to support the efficacy of thrombectomy within 24 h for BAO, demonstrating significantly better treatment safety and efficacy (43). Two subsequent trials, the BASICS (BASilar artery International Cooperation Study) and the BEST (Endovascular Treatment vs. Standard Medical Treatment for Vertebrobasilar Artery Occlusion) investigated thrombectomy for BAOs in patients presenting within 6 h and 8 h of stroke, respectively (Table 3) (44, 45). BASICS randomized patients into the thrombectomy and best medical therapy arms, with slightly better functional outcomes seen in the thrombectomy group (44.2% vs. 37.7%) and lower mortality rates (38.4% vs. 43.2%), but with higher sICH rates (4.5% vs. 0.7%). Patients who demonstrated higher rates of actual or potential favorable 90-day mRS scores (mRS 0–3) after thrombectomy were those who presented with moderate (NIHSS 10–19) and severe (NIHSS ≥20) neurological deficit. A case of a 78-year-old female with NIHSS score of 28 due to an upper basilar occlusion, successfully treated with the SOLUMBRA technique achieving TICI 3 recanalization, is presented in Figure 3. Similarly, BEST compared thrombectomy to best medical therapy but was terminated early due to poor recruitment and high treatment crossover rates (22% from best medical therapy to thrombectomy). 47% of patients treated with thrombectomy reported higher favorable 90-day mRS scores (mRS 0–3), compared to 24% of control. Overall, BASICS and BEST did not demonstrate superiority of thrombectomy over best medical treatment, which were addressed by subsequent trials.

**Table 3:** Characteristics of RCTs investigating mechanical thrombectomy in basilar artery occlusion.

Trial	BASICS (42)	BEST (43)	ATTENTION (44)	BAOCHE (45)
Study characteristics				
Publication year	2021	2020	2022	2022
Enrolment period	2011–2019	2015–2017	2021–2022	2016–2022
Country	Multi-national (7 countries)	China	China	China
Patients	300	131	340	217
Inclusion criteria				
Age	Initially 18–85, later expanded to ≥18	≥18	≥18	18–80
Time	0–6 h	0–8 h	0–12 h	6–24
Vessels	BA (proximal, middle, and distal)	VA (V4), BA	VA (V4), BA (proximal, middle, and distal)	BA (Proximal, middle, and distal)
pc-ASPECTS	≥10, later expanded to <10	–	≥6 for age <80 ≥8 for age ≥80	≥10, later expanded to ≥6 and pons-midbrain index ≤2
NIHSS	Initially ≥10 but removed after 91 patients enrolled	None	≥10	≥10 initially, changed to ≥6 after 84 patients enrolled
Baseline mRS	0–2	0–2	0–2 (age ≤80); 0 (mRS >80)	0–1
IV tPA eligibility	Not required	Not required	Not required	Not required
Outcomes				
mTICI 2b, 3	72%	71%	93.3%	88.1%
mRS 0–2	35% vs. 30%	33% vs. 28%	33.2% vs. 10.5%	39% vs 14%
mRS 0–3	44% vs. 37.7%	47% vs. 24%	46 vs. 22.8%	46.4% vs. 24.3%
Mortality	38.3% vs. 43.2%	33% vs. 38%	36.7% vs. 55.3%	30.9% vs. 42.1%
sICH	3.9% vs. 0.7%	8% vs. 0%	5.3% vs. 0%	5.9% vs. 1.1%
Procedure-related complications	3%	–	14%	11%

ASPECTS, Alberta Stroke Program Early CT Score; BA, basilar artery; mRS, modified Rankin score; mTICI, modified thrombolysis in cerebral infarction score; NIHSS, National Institutes of Stroke Scale; NNT, number needed to treat; sICH, symptomatic intracranial bleeding; TPA, tissue plasminogen activator; V4, V4 segment of the vertebral artery.

ATTENTION (Endovascular Treatment for Acute Basilar Artery Occlusion) is a Chinese trial that that randomized thrombectomy and medical treatment for patients with BAO presenting within 12 h of stroke, with baseline NIHSS score > 10, and age-adjusted pc–ASPECTS (>6 points for patients aged <80; > 8 points for those aged > 80 (46). Additionally, 40% of patients in the thrombectomy group underwent angioplasty and stenting. A significantly higher proportion of patients in the thrombectomy demonstrated good functional outcome (mRS 0–3) compared to control (46% vs. 23%). The Basilar Artery Chinese Endovascular Trial (BAOCHE) is another RCT that investigated the safety and efficacy of thrombectomy in the late time window (6–24 h), with NIHSS ≥ 6, and pc–ASPECTS <6, and pons-midbrain index ≤2 (47). Like ATTENTION, additional angioplasty or stenting was performed in 55% of thrombectomy-treated patients. The thrombectomy group demonstrated higher rates of favorable functional outcomes (46.4% mRS 0–3 vs. 24.3%), lower mortality rates (31% vs. 42%), but with increased rates of sICH (6% vs. 1%). The etiology of BAO is also important given the Chinese population recruited in both studies, who are likely to have a significant intracranial atherosclerosis burden (26).

### 2.4 Tandem occlusions

Tandem occlusions are defined as concurrent intracranial LVOs and extracranial internal carotid artery (ICA) occlusion or severe stenosis. With an incidence of 10%–20% of anterior circulation LVO strokes (48), endovascular treatment of tandem occlusions is clinically and technically challenging, requiring strict management of blood pressure, balancing thrombotic and bleeding risks, and treatment of both extracranial and intracranial lesions. Tandem occlusions are primarily caused by carotid artery atherosclerosis or, less commonly, acute dissection (49). The lack of efficacy of IVT in tandem occlusions has shifted treatment focus to thrombectomy to achieve sufficient recanalization in these patients (50).

**Figure 4:**
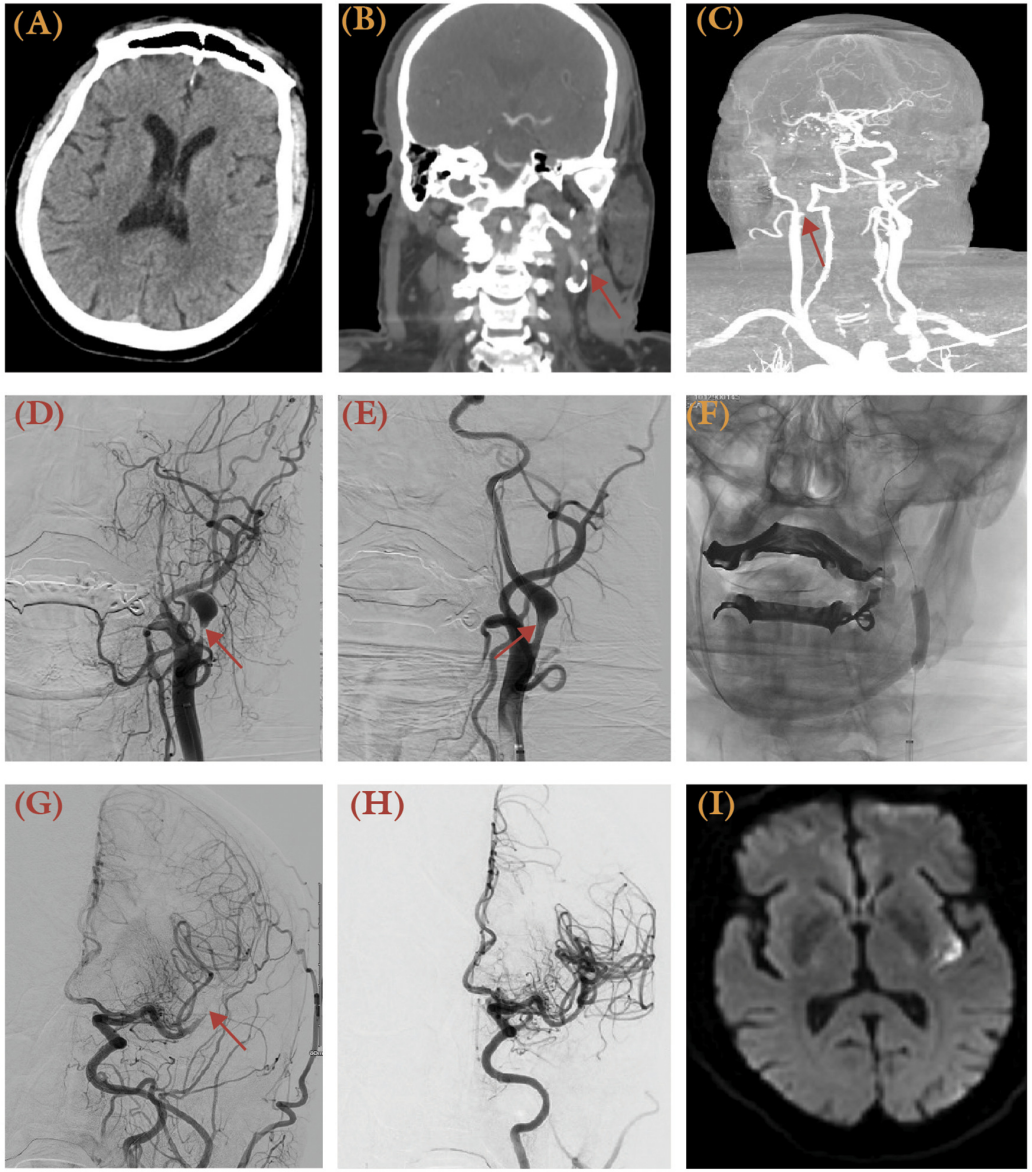
A 75-year-old male a history of hypertension presented with altered mental status and aphasia, with NIHSS 14 and last known well 2 h prior to presentation. **(A)** Brain CT revealed no apparent hypodensity of the brain parenchymal. **(B,C)** CTA revealed atherosclerotic plaque resulting in severe stenosis within the left carotid bifurcation. AP view of digital subtraction angiography (DSA) revealed severe stenosis at the origin of the left ICA following a left CCA injection before **(D)** and after angioplasty **(E)**, with improved caliber of the ICA (arrow). **(F)** AP view of unsubtracted x-ray illustrates the balloon used for angioplasty (arrows). **(G)** AP view angiography of the left CCA injection demonstrated an occlusion over the left M2 (arrow). **(H)** Following mechanical thrombectomy with aspiration technique, a TICI 3 reperfusion was achieved. **(I)** Despite successful recanalization, post-procedural MRI confirmed a small left posterior insular cortex infarct.

Various thrombectomy strategies have been proposed for tandem occlusions, including antegrade approaches (treating the extracranial lesion first), retrograde approaches (treating the intracranial LVO first), balloon angioplasty, and conservative thrombectomy without extracranial intervention. Meta-analyses and registry data indicate no significant differences in outcomes between antegrade and retrograde approaches, though the retrograde method may offer faster recanalization (51, 52). Acute carotid stenting, while effective in restoring blood flow and reducing stroke recurrence, requires immediate initiation of antiplatelet therapy, increasing the risk of hemorrhagic complications. Despite these risks, stenting has demonstrated improved odds of functional independence and successful reperfusion in multiple studies, and patient selection should consider factors like underlying etiology, core infarct volume, and patient anatomy (53). A case of a 75-year-old male with NIHSS score of 14 and severe stenosis within left ICA bifurcation, treated with angioplasty and aspiration achieving TICI 3 reperfusion, is illustrated in Figure 4.

Emerging evidence supports the overall benefit of thrombectomy for tandem occlusions. A meta-analysis of 1,373 patients demonstrated improved functional outcomes at 90 days (OR 1.43) (54), while pooled data from the ETIS and TITAN registries highlighted better reperfusion and favorable outcomes with stenting, although with higher rates of non-symptomatic intracranial hemorrhage (55). However, clinical benefit was less pronounced in tandem occlusions secondary to dissection, emphasizing the need for personalized decision-making based on etiology. Ongoing randomized trials, such as DST-TANDEM, EASI-TOC, and TITAN, aim to provide more definitive guidance on optimal management strategies for tandem occlusions (56–58). These studies are expected to clarify the benefits of acute stenting and refine patient selection criteria.

## 3 Personalization by lesion core and infarct size

### 3.1 Large core infarcts

Large core infarcts are strokes identified through neuroimaging as those with extensive areas of brain tissue injury. The Alberta Stroke Program Early CT (ASPECTS) score characterizes stroke infarct burden, with earlier stroke trials focusing on small preprocedural infarct sizes, defined as ASPECTS 6–10 (9, 11). The stroke core volume on CT perfusion (CTP) or MRI also defines infarct burden, with the DAWN and DIFUSE-3 trials selecting patients with median core volumes of 7.6 and 9.4 ml, respectively (11, 59). Infarct burden is associated with outcomes after thrombectomy, with a favorable likelihood significantly decreasing with each ASPECT score (60). Accordingly, upper limits of 50–70 ml core volume or ASPECTS of 6 were set in earlier trials to reflect this (28).

The RESCUE-Japan LIMIT was the first trial to randomize patients with large core infarcts for mechanical thrombectomy and best medical treatment (Table 4) (61). Selecting patients with ASPECTS 3–5, thrombectomy significantly improved functional independence, with 31% of patients achieving 90-day mRS 0–3 scores compared to 12.7% in the best medical treatment group. Similarly, the SELECT 2 trial defined large infarcts as ASPECTS 3–5 and stroke volume ≥50 ml, with 20.7% of thrombectomy-treated patients achieving an mRS score of 0–2 compared to 7% in the medical treatment group (62). The ANGEL-ASPECT trial also supported these findings, with large infarcts defined as ASPECTS 3–5 and ASPECTS 0–2 or ≥5 with infarct volumes 70–100 ml (63). This trial showed that 30% of thrombectomy patients achieved mRS 0–2 compared to 11.6% in the medical group. National stroke guidelines now support thrombectomy for select patients with ASPECTS 3–5, recognizing the potential for improved outcomes and cost-effectiveness in this population (64). While these trials confirmed the benefits of thrombectomy, the challenges in treating large infarct strokes with thrombectomy are well recognized. These patients are at increased risk for hemorrhagic transformation, cerebral edema, prolonged hospitalization, and mortality (53). In contrast, thrombectomy may reduce the need for decompressive hemicraniectomy by preventing malignant cerebral edema, potentially improving both survival and quality of life (65).

**Table 4:** Characteristics of RCTs investigating mechanical thrombectomy in large core infarcts.

Trial	RESCUE-JAPAN LIMIT (59)	SELECT 2 (60)	ANGEL-ASPECT (61)	TENSION (64)	TESLA (65)	LASTE (66)
Study characteristics						
Publication year	2022	2023	2023	2023	2024	2024
Enrolment year	2018–2020	2019–2022	2020–2022	2018–2023	2019–2022	2019–2022
Country	Japan	Multi-national	China	Europe and Canada	USA	France and Spain
Patients	203	352	456	253	300	333
Inclusion criteria						
Age	≥18	18–85	18–80	≥18	18–85	≥18
Time	0–24 h	0–24 h	0–24 h	0–11 h	0–24 h	0–6.5 h
Vessels	ICA, M1	ICA, M1	ICA, M1	ICA, M1	ICA, M1	ICA, M1
ASPECTS/Lesion volume	ASPECTS 3–5	ASPECTS 3–5; infarct core volume ≥50 ml	ASPECTS 3–5; ASPECTS < 3 with infarct core 70–100 ml; ASPECTS >5 with volume 70–100 ml	3–5	2–5	0–5 (4–5 for patients ≥80 years)
NIHSS	≥6	≥6	6–30	<26	≥6	≥6
Baseline mRS	0–1	0–1	0–1	0–2	0–1	0–1
TPA eligibility	Not required	Not required	Not required	Not required	Not required	Not required
Outcomes						
mRS 0–2	14% vs. 7.8%	20% vs. 7%	30% vs. 12%	17% vs. 2%	14.6% vs. 8.9%	13.3% vs. 4.9
mRS 0–3	31% vs. 12.7%	38% vs. 19%	47% vs. 33%	31% vs. 13%	29.8% vs. 19.9%	–
Mortality	18% vs. 23.5%	38% vs. 42%	22% vs. 20%	40% vs. 51%	35% vs. 33%	36.1% vs. 55.5%
sICH	9% vs. 4.9%	0.6% vs. 1.1%	6.1% vs. 2.6%	5% vs. 5%	4% vs 1.3%	9.6% vs. 5.7%

ASPECTS, Alberta Stroke Program Early CT Score; ICA, internal carotid artery; M1, M1 segment of the middle cerebral artery (MCA); mRS, modified Rankin score; mTICI, modified thrombolysis in cerebral infarction score; NIHSS, National Institutes of Stroke Scale; NNT, number needed to treat; sICH, symptomatic intracranial bleeding; TPA, tissue plasminogen activator.

The Thrombectomy in Stroke with Extended Lesion and Extended Time Window (TENSION) trial, randomizing patients with ASPECTS 3–5 within 12 h of onset, was halted early due to significantly better mRS scores and lower mortality rates in the thrombectomy group, with comparable sICH rates between the two treatment arms (66). Recently, the TESLA and LASTE trials have demonstrated a holistic approach in thrombectomy that considers salvageable penumbra, infarct location, and patient-specific factors, in addition to lesion volume. TESLA evaluated thrombectomy in patients with ASPECTS 2–5 within 24 h of stroke onset (67). While the 90-day mean-utility mRS score favored thrombectomy (2.93 vs. 2.27 in the control group), the adjusted difference did not meet statistical significance. Mortality rates were similar between groups (35.3% vs. 33.35%), but higher rates of sICH (4.0% vs. 1.3%) and other hemorrhagic complications were noted in the thrombectomy group. LASTE demonstrated that thrombectomy benefits patients with large infarcts regardless of size, including patients with ASPECTS ≤5 and no minimum ASPECTS limit (68). Among patients with a median ASPECTS of 2, thrombectomy improved 90-day functional outcomes (OR 1.63; 95% CI 1.29–2.06; NNT = 4) and reduced all-cause mortality (36.1% vs. 55.5%). Benefits extended to quality of life at 90 and 180 days and reduced the rate of decompressive craniectomy. Like TESTLA, thrombectomy was associated with higher rates of sICH (9.6% vs. 5.7%) and procedural complications (6.9%).

With some patients with large infarcts achieving favorable recovery, while others with smaller infarcts may have poor prognoses, careful patient selection based on holistic considerations rather than infarct size alone is pertinent to guide therapy (69). The ratio of salvageable penumbra to infarct core size is critical in determining patient eligibility and potential benefit. Infarct location also plays a role, with the involvement of eloquent brain tissue potentially impacting outcomes significantly (70). Younger patients often also show better recovery potential, making them more likely to benefit from thrombectomy even with larger infarct burdens.

### 3.2 Small core infarcts

Small core infarcts, characterized by minimal established ischemic damage, offer a larger penumbral region for potential salvage, making them prime candidates for thrombectomy (71). The ESCAPE, REVASCAT, SWIFT PRIME, and EXTEND-IA trials, combined in the HERMES meta-analysis, demonstrated the significant benefit of thrombectomy in patients with small core infarcts, as identified by high ASPECTS scores on non-contrast CT or favorable perfusion imaging using software like RAPID (5–9). Across these studies, thrombectomy significantly improved functional outcomes, with a strikingly low NNT of 2.6 for one-point mRS improvement and 5 for achieving functional independence (mRS 0–2). In extended time windows (>6 h), trials like DAWN and DEFUSE 3 reaffirmed the efficacy of thrombectomy in late presenters with small core infarcts and favorable penumbral patterns, selected through advanced neuroimaging (11, 12). Remarkably, these trials showed no significant increase in sICH between thrombectomy and control groups, reinforcing the safety of thrombectomy when patient selection is rigorously guided by imaging. Retrospective studies have also shown that simplified imaging strategies, such as multiphase CTA, can reliably identify patients with good collaterals and small infarct volumes eligible for thrombectomy beyond 6 h, with comparable outcomes to more advanced imaging modalities (72, 73).

## 4 Personalization by patient-specific characteristics

### 4.1 Patients with low NIHSS scores

The National Institutes of Health Stroke Scale (NIHSS) is an 11-category tool designed to quantify neurological deficits in stroke patients (74). While and NIHSS score ≥6 is typically associated with more severe strokes and is often an inclusion criterion for mechanical thrombectomy trials, patients with NIHSS scores 0–5 comprise a unique subset with distinct challenges and opportunities for treatment personalization (75). Low NIHSS scores are observed in 4%–8% of patients with minor neurological deficits despite harboring an LVO, with these patients making up 10% of all LVO strokes undergoing thrombectomy (76, 77). Importantly, ≥50% of these patients present beyond six hours from symptom onset and up to 35% of these patients may have unfavorable outcomes at 90 days, complicating treatment decisions (28). A key challenge in managing low NIHSS stroke patients it the risk of neurologic deterioration, defined as an NIHSS increase in ≥2 points (78). Proximal occlusions, larger thrombus burdens, and failure of collateral blood flow are predictors of this decline, which is associated with poor patient outcomes (79).

As low NIHSS patients have been historically excluded from most thrombectomy trials, evidence for mechanical thrombectomy in this group is limited to observational studies and case series, with no RCTs definitively establishing efficacy. Retrospective analyses and meta-analyses have yielded mixed results. While some studies suggest thrombectomy may confer benefits in select low NIHSS patients, others report no significant differences compared to best medical therapy, with thrombectomy associated with higher risks of sICH in some cases (80–83). Personalization of treatment for low NIHSS patients involve appropriate patients, which may comprise higher-risk subgroups like those with proximal occlusions, larger perfusion deficits, or symptoms affecting motor or language function (28).

While some retrospective studies suggest that immediate thrombectomy may lead to better outcomes than rescue therapy following neurological deterioration in this subgroup of patients, the heterogeneity of low NIHSS stroke patients necessitates further research to define optimal selection criteria. Current guidelines recommend enrolling low NIHSS patients with LVO into RCTs (25), which ongoing trials like ENDOLOW (Endovascular Therapy for Low NIHSS Ischemic Strokes) and MOSTE (Minor Stroke Therapy Evaluation) aim to address the knowledge gaps in this area (84).

### 4.2 Older patients—octogenarians and nonagenarians

The role of patient age in stroke thrombectomy is a critical area for personalization, particularly in older populations such as octogenarians and nonagenarians. These groups often present unique challenges due to a higher prevalence of comorbidities, disabilities, and frailty, as well as lower brain reserve, which can influence both the risks and benefits of thrombectomy (85).

While most thrombectomy trials have not excluded older adults, the average age of participants included is 68 years, with only 15% over the age of 80 (9). Despite these limitations, growing data suggest that thrombectomy can be both technically successful and clinically beneficial for select older patients (86). Furthermore, studies have shown that older patients can achieve comparable success rates to younger patients, provided that procedural challenges such as increased vessel tortuosity, which can complicate arterial access, are effectively managed (87).

An important association with thrombectomy outcomes for older patients is their baseline functional status. Studies indicate that nonagenarians with a baseline mRS score ≤2 are more likely to achieve favorable outcomes after thrombectomy compared to those with higher mRS scores. For instance, premorbidly independent nonagenarians have demonstrated favorable outcomes in up to 39% of cases, with some studies reporting similar or even better results compared to octogenarians (85, 88). However, patients with significant pre-stroke disabilities (e.g., mRS >2) often experience poorer outcomes, though exceptions may exist for otherwise healthy individuals with orthopedic or non-cognitive impairments (89). While overall outcomes for older patients are generally poorer compared to younger populations, thrombectomy has been shown to provide meaningful benefits (90). Nonagenarians who achieve successful reperfusion demonstrate greater early neurological recovery, reduced mortality, and improved 90-day functional outcomes compared to those managed with thrombolytic therapy alone (89). However, the range of favorable outcomes is wide, with rates reported between 12% and 31% (91, 92).

The decision to pursue thrombectomy in older patients should be individualized, considering baseline functional independence, comorbidities, and patient-specific goals of care. Currently, the European Stroke Association guidelines recommend thrombectomy for octogenarians with appropriate imaging characteristics, although the case for nonagenarians is not specifically addressed (10, 93). This highlights the importance of a holistic approach to decision-making, including the evaluating imaging findings, the risk of procedural complications, and potential benefits in restoring independence.

### 4.3 Patient-specific biomarkers

The paradigm of personalization of stroke thrombectomy has increasingly shifted to understanding the predictive potential of patient-specific biochemical, inflammatory, and physiological markers in optimizing outcomes and minimizing risks. High blood glucose levels, markers of renal health (e.g., low serum uric acid to creatinine ratio), and high C-reactive protein have been found to be predictors of hemorrhagic transformation and poor outcomes post-thrombectomy (94–96). The TICI-ASPECTS–glucose (TAG) score, representing the combined effects of moderate to low recanalization, low ASPECTS, and hyperglycemia has been validated for predicting sICH post-thrombectomy, with a 50% increased sICH likelihood per-point increase in TAG score (97). Baseline glucose, in addition to increasing age, baseline NIHSS, onset to puncture >6 h, sICH, and pneumonia were also found to be independent predictors of poor functional outcomes post-thrombectomy in a Chinese population (98). The role of systemic inflammation in stroke recovery and thrombectomy outcomes is also well documented. Inflammatory markers like the blood cell ratios and indices of systemic inflammation have been linked to functional outcomes, sICH, stroke-associated pneumonia, and mortality rates post-thrombectomy. For example, higher platelet-to-neutrophil (PNR) and lower platelet-to-lymphocyte (PLR) ratios were associated with favorable 3-month outcomes post-thrombectomy, with optimal cutoff values of PNR >41 and PLR <145 (99). Similarly, patients with a high systemic immune-inflammatory index (SII) were more likely to develop malignant cerebral edema, which accounted for 40.3% of the poorer prognosis among these individuals (100), while systemic inflammatory response index (SIRI) has demonstrated predictive value for stroke-associated pneumonia post-thrombectomy (101). Blood pressure variability (BPV) and other hemodynamic factors also impact thrombectomy outcomes. Increased pulse pressure variability (PPV), a parameter of BPV, in the first 24 h post-thrombectomy strongly predicts 90-day functional outcomes, with adjusted ORs exceeding 40 for the highest PPV ranges. Similarly, deviations from a linear systolic blood pressure course, such as failing to maintain a gradual decline from 130 to 123 mmHg over 24 h, were associated with a 47% reduced likelihood of functional independence (102). High-resolution BP monitoring also suggests that successive variation in systolic BP over 5-minute intervals better predicts outcomes compared to static BP measures (AUC 0.74, *p* = 0.031) (103). The DETERMINE trial is exploring whether personalized BP management, defined as maintaining MAP within 10% of baseline, leads to better functional outcomes compared to the standard target range of 140–180 mmHg (104). Preliminary observational studies support this approach, as patients who spent more time outside their personalized autoregulatory BP limits showed worse 90-day outcomes, with odds of poor outcomes increasing by 1.84 for every 10% of time spent above the upper autoregulatory limit. Notably, hemorrhagic transformation was more frequent when mean arterial pressure exceeded these limits (16.0% vs. 10.9%, *p* = 0.042) (105).

Patient clinical, demographic, and biomarker data can be integrated into predictive algorithms to enhance personalized thrombectomy. Machine learning (ML) algorithms like random forest (RF), extreme gradient boosting (XGBoost), and neural networks have demonstrated improved stroke detection, thrombectomy candidacy, and prognosis. An RF model, using 35 clinical and demographic variables, was able to predict the presence of LVOs and appropriately thrombectomy candidates with AUCs of 0.91 and 0.93, respectively, outperforming conventional stroke scales (106). By integrating preoperative and postoperative variables, such as NIHSS scores, an XGBoost model was able to improve predictive ability for unfavorable 3-month outcomes, enabling timely therapeutic adjustments (107). Additionally, a Back propagation neural network was able to predict 3-month mRS post-thrombectomy using patient clinical data, achieving prediction accuracy, sensitivity, and specificity of 96.1%, 98.3%, and 87.5%, respectively (108). The utility of ML applications in personalized stroke thrombectomy has been confirmed by a recent meta-analysis, which pooled five studies in predicting favorable 90–day functional outcomes (mRS 0–2) (109). With overall AUC of 0.85, sensitivity of 0.80, specificity of 0.78, and diagnostic odds of 12.6, the efficacy of ML-based predictive value holds great potential for optimizing patient identification for thrombectomy.

### 4.4 Special patient populations

Special populations refer to patient groups who are often excluded from major clinical trials, including children, patients with hematologic abnormalities, active cancer, recent surgery, collagen vascular diseases, or pregnant women. Although case series have demonstrated the safety and feasibility of thrombectomy in many of these populations, these cases are rare, and more studies are necessary to strengthen the evidence base of precision thrombectomy care (28). Guidelines support personalized thrombectomy in neonates, infants, children, or adolescents, recommending standard radiation dose-minimization techniques for diagnostic and therapeutic procedures, and non-ionizing imaging for initial stroke evaluation in children under 10 years of age. A thrombocytopenic status should not preclude thrombectomy, although hematologic consultation and, in some cases, platelet transfusion may be required for extremely thrombocytopenic patients (<20,000/mm^3^ platelet count). Female patients should be offered thrombectomy regardless of pregnancy status, with proper focus on appropriate patient selection, radiation safety protocols, and multidisciplinary team input where necessary. Personalized thrombectomy should be also offered in patients at high risk of diagnostic and therapeutic complications, including those with active endocarditis, collagen vascular disease, and recent surgical history. These patient groups will benefit from prioritization of non-invasive imaging where appropriate, careful procedural planning, and multidisciplinary input to mitigate risks of procedural planning or hemorrhagic transformation (110).

## 5 Personalization by devices and approaches

### 5.1 Personalization through thrombectomy devices

Mechanical thrombectomy restores cerebral blood flow in patients with acute ischemic stroke using stent retrievers, aspiration catheters, or a combination of these devices to remove the thrombus under fluoroscopic guidance. Device selection and procedural techniques have evolved significantly, driven by innovations in catheter systems, guidewires, and thrombectomy devices, which have enhanced safety, efficacy, and procedural speed.

Stent retrievers are among the earliest and most established devices for thrombectomy. These devices are introduced through a microcatheter, deployed at the site of occlusion, and used to capture and remove the thrombus by retracting it along the vessel wall. Stent retrievers can be personalized according to occlusion site and clot characteristics. More recent innovations of stent retrievers are optimized for distal vessel occlusions with vessel diameters 0.5–2 mm (111, 112). Stent retrievers are also preferred for soft, red-blood-cell-rich thrombi, demonstrating higher recanalization in these lesions (113). While stent retrievers are effective, challenges such as vessel tortuosity and clot stiffness can reduce their efficacy. Additionally, dragging the device across the endothelium may cause vascular damage (114), a limitation addressed by emerging techniques combining stent retrievers with aspiration catheters.

Aspiration catheters remove thrombi by creating negative pressure through a syringe or pump (115). This approach, typified by the ADAPT technique, is simpler than stent retriever thrombectomy and often results in less endothelial damage (116). Larger bore catheters, such as the Trac Star 088 and Millipede 088, have improved the first-pass effect (FPE) and revascularization rates by maximizing suction force and clot capture. Small diameter aspiration catheters have also been developed for distal vessel occlusion (117). Like stent retrievers, the efficacy of aspiration catheters also depends heavily on clot characteristics, demonstrating efficacy in soft, fibrin-rich clots (118). Aspiration catheters also favor lesions where the angle between the catheter and the clot is greater than 125.5° (119). Aspiration is particularly advantageous in reducing procedural time and complications like embolization to new territories. It is also associated with less endothelial damage (116).

Combined techniques utilizing both stent retrievers and aspiration catheters have emerged to address the limitations of individual devices. Techniques like Solumbra and SAVE (Stent Retriever Assisted Vacuum-locked Extraction) capture the thrombus between the stent and catheter, enhancing recanalization rates (120, 121). The ASTER (Contact Aspiration vs. Stent Retriever for Successful Revascularization) and COMPASS (Comparison of Direct Aspiration vs. Stent Retriever as a First Approach) trials found no significant difference in outcomes between stent retrievers and aspiration used as standalone strategies (122, 123). Similarly, the ASTER2 trial did not show a significant improvement in final near-complete or complete reperfusion (eTICI 2c/3) with the combined approach combined to stent retriever alone, though the combination did result in higher early successful reperfusion after the first device pass (124). The VECTOR trial further found no significant advantage of the combined technique over contact aspiration alone in patients with susceptibility vessel sign-positive occlusions (125). Finally, the Separator-3D trial demonstrated noninferiority of a 3D stent retriever with aspiration compared to aspiration alone, with no significant differences in clinical or angiographic outcomes (126).

Combined techniques may still offer practical benefits in certain clinical contexts, such as clot heterogeneity and vessel tortousity (127). The addition of balloon guide catheters (BGCs) to these techniques has traditionally been thought to improve procedural outcomes and reduce embolic complications (128). However, recent evidence from the PROTECT-MT trial raises concerns about their use (129). In this large multicenter RCT, BGC use was associated with significantly worse 90-day functional outcomes and a higher, though not statistically significant, rate of mortality compared to conventional guide catheters. These results challenge previous assumptions and highlight the need for further studies to clarify the role of BGCs in modern thrombectomy workflows.

### 5.2 Personalization through thrombectomy approaches

While transfemoral arterial access (TFA) is the standard approach, transradial access (TRA) or direct carotid puncture can be employed in specific cases. TRA is becoming increasingly popular among neurointerventionalists due to its reduced rate of access site complications, improved patient satisfaction, earlier ambulation, and shorter hospitalization (130, 131). However, this approach is technically difficult, requiring a steeper learning curve and specific patient anatomical patterns to ensure procedural success (132, 133). Early case series have demonstrated the safety and feasibility of TRA for anterior circulation LVOs, with results comparable to conventional transfemoral systems (134). A comprehensive meta-analysis involving 13 studies and 4,759 patients has also confirmed safety and efficacy of TRA in acute ischemic stroke, revealing comparable recanalization rates, first pass reperfusion rates, mean number of passes, mortality and sICH rates, and post-treatment mRS and NIHSS scores to TFA (135). TRA is particularly suited for patients with challenging vascular anatomy, including those with a high B.A.D. (Bovine arch variant, Aortic arch type, and ICA Dolichoarteriopathy) score (136, 137). The B.A.D. is a composite score that assigns points based on the presence of a bovine arch (1 point), aortic arch type II (1 point) or III (2 points), and ipsilateral ICA tortuosity or coiling (1 point), with higher scores (≥2) indicating more difficult access (137). TRA may also benefit patients with complex comorbidities, prior vascular procedures, and older age (131, 138). While the growing evidence supports TRA as a viable alternative in appropriate patient groups, more studies and integrative radial-specific catheter development are necessary to establish broader consensus and widespread adoption.

When TFA or TRA fail due to anatomical constraints, direct carotid puncture serves as a rescue strategy in improving angiographic and functional outcomes (139). Direct carotid puncture allows direct access to the occlusion site, bypassing tortuous or diseased vessels. This technique allows direct access to the occlusion site, favoring vessels with high B.A.D. scores and patients with co-existing vascular pathologies (137, 140). Although this technique can result in complications of the punctured vessel, thromboembolic events, and airway compromise, only 1%–2% of affected patients will require re-intervention (141).

## 6 Conclusions

Through advances in neurovascular imaging and developments in thrombectomy devices and approaches, thrombectomy has revolutionized AIS treatment, with current evidence demonstrating a shift to personalized approaches to optimize outcomes. Through careful patient selection that incorporates patient-specific, lesion-specific, and biomarker-guided strategies, the paradigm shift in stroke thrombectomy has expanded patient eligibility criteria, allowing safe and effective interventions for previously excluded groups. The increase in patient load requires appropriate distribution of the neurovascular workforce to ensure equitable access to stroke thrombectomy. However, challenges persist in refining patient selection, with ongoing trials aiming to inform treatment decisions whilst balancing treatment-related risks. Further studies are warranted to refine the role of patient-specific biomarkers, incorporating emerging tools like artificial intelligence to enhance stroke management.
